# Dynamic spillovers between the term structure of interest rates, bitcoin, and safe-haven currencies

**DOI:** 10.1186/s40854-021-00274-w

**Published:** 2021-08-02

**Authors:** David Y. Aharon, Zaghum Umar, Xuan Vinh Vo

**Affiliations:** 1 Department of Business Administration, Ono Academic College, Zahal 104 Street, 5545173 Kiryat Ono, Israel; 2 College of Business, Zayed University, Abu Dhabi, UAE; 3 Institute of Business Research, University of Economics Ho Chi Minh City, Ho Chi Minh City, Vietnam

**Keywords:** Bitcoin, Term structure slope, Curvature, Diebold and Yilmaz, Connectedness, Cryptocurrency, Forex, Currencies, Safe haven, C53, E43, F36, G12, G17

## Abstract

This study examines the connectedness between the US yield curve components (i.e., level, slope, and curvature), exchange rates, and the historical volatility of the exchange rates of the main safe-haven fiat currencies (Canada, Switzerland, EURO, Japan, and the UK) and the leading cryptocurrency, the Bitcoin. Results of the static analysis show that the level and slope of the yield curve are net transmitters of shocks to both the exchange rate and its volatility. The exchange rate of the Euro and the volatility of the Euro and the Canadian dollar exchange rate are net transmitters of shocks. Meanwhile, the curvature of the yield curve and the Japanese Yen, Swiss Franc, and British Pound act mainly as net receivers. Our static connectedness analysis shows that Bitcoin is mainly independent of shocks from the yield curve’s level, slope, and curvature, and from any main currency investigated. These findings hint that Bitcoin might provide hedging benefits. However, similar to the static analysis, our dynamic analysis shows that during different periods and particularly in stressful times, Bitcoin is far from being isolated from other currencies or the yield curve components. The dynamic analysis allows us to observe Bitcoin’s connectedness in times of stress. Evidence supporting this contention is the substantially increased connectedness due to policy shocks, political uncertainty, and systemic crisis, implying no empirical support for Bitcoin’s safe-haven property during stress times. The increased connectedness in the dynamic analysis compared with the static approach implies that in normal times and especially in stressful times, Bitcoin has the property of a diversifier. The results may have important implications for investors and policymakers regarding their risk monitoring and their assets allocation and investment strategies.

## Introduction

The outbreak of the COVID-19 pandemic in early 2020 reinvigorated the search for useful risk management, hedging strategies, and investors’ demand for safe-haven assets. Although traditionally major currencies have been regarded as safe-haven assets, several financial market downturns, such as the 2008 subprime crisis and the 2011 sovereign debt crisis, raised doubts about their diversification benefits in turbulent times. Demir et al. (2018) identified 2008 as a major turning point, when the public’s distrust in the traditional financial system led to the quest for a neutral currency, independent of governments or monetary policy. This Promised Land vision created Bitcoin and other cryptocurrencies. Bitcoin’s price has recently soared to record highs, thereby attracting more attention from investors, financial market participants, and policymakers.^1^

The sovereign yield curve is one of the most recognized and familiar components of a centralized monetary system, which is also a major economic indicator, advocated by both academicians and practitioners. Previous studies (e.g., Harvey 1988; Ang et al. 2006; Wheelock and Wohar 2009; Riaz et al. 2020; Umar et al. 2021a, b) have documented that the information embedded in the yield curve can assist investors in predicting future economic fundamentals. In addition, interest rates are commonly recognized as a monetary tool for shaping forex exchange rates. Accordingly, studies have documented the theoretical and practical channels through which the yield curve may affect the fluctuations in currencies (e.g., Chen and Tsang 2013; Jotikasthira et al. 2015; Baek and Lee 2020).

However, to the best of our knowledge, the connectedness dynamics among the term structure of interest rates, a decentralized currency (e.g., Bitcoin), and the traditional safe-haven currencies are yet to be explored. Inspired by the growing stream of literature focused on the connectedness of various financial and economic variables, the current study analyzes the joint and pairwise connectedness of the yield curve components, Bitcoin, and five major safe-haven currencies (the Canadian dollar [CAD], the Euro, the Japanese Yen [JPY], the Swiss Franc [CHF], and the British Pound Sterling [GBP]). We explore their connectedness in a static framework and under different market conditions in a dynamic time-varying framework. The findings may enhance our understanding of the growing debate about the role of Bitcoin and major currencies in the context of portfolio management and the impact of yield curve movements in shaping their behavior. Given the growing interest in cryptocurrencies by investors, firms, and financial market participants globally, and their potential impact on market stability, we maintain that this is a timely investigation.

To accomplish the aims of this study, we retrieve the daily historical data of the US terms structure yields, exchange rates, and historical volatility of the exchange rates for Bitcoin, Euro, JPY, CAD, CHF, and GBP. Our sample covers the period from May 11, 2010, to November 26, 2020, based on the availability of the matched data series. We estimate the US yield curve components using Diebold and Li’s (2006) modification of Nelson and Siegel’s (1987) model. We then measure the connectedness among the system variables, using the novel approach of Diebold and Yilmaz (2009, 2012, 2014). The examination allows us to map their dynamic role in terms of risk transmitters versus receivers.

Our study contributes to several evolving strands in the literature. First, we add to the studies dealing with the role of traditional safe-haven currencies in the framework of diversification and risk reduction under market stress (e.g., Fatum and Yamamoto 2016; Dao et al. 2019; Urquhart and Zhang 2019). We analyze the connectedness of the commonly used safe-haven currencies with all the components of the sovereign yield curve and the most dominant cryptocurrency, Bitcoin. Several public companies are now accepting Bitcoin, and such firms operate internationally using different exchange rates. Thus, analyzing whether Bitcoin can be a safe shelter against other currencies in stressful times is worthwhile.

Second, our examination also sheds light on how conventional currencies interact with Bitcoin. This examination is critical given that Bitcoin has become increasingly popular and most firms are planning to accept it as payment for their sales.

Third, we extend the debate in the literature regarding the place of cryptocurrencies in different types of asset classes. Although this line of research is rapidly evolving, there is no unanimity regarding their role, particularly for Bitcoin. Several studies have suggested that cryptocurrencies have a safe-haven property (e.g., Zhang and Wang, 2020). However, others conclude the contrary (e.g., Smales 2019; Conlon and McGee 2020), creating a controversial debate.

Fourth, cryptocurrencies have been primarily designed to be insulated and independent from any traditional government monetary policy system. Hence, our study can shed light on Bitcoin’s connectedness with the yield curve. This examination is particularly important in helping policymakers in various countries decide whether to integrate Bitcoin or any other cryptocurrency into their settlement systems. It will also help financial markets designers seeking to maintain trading stability. To the best of our knowledge, this is the first study to employ a dynamic connectedness approach relating Bitcoin to all three components of the entire yield curve (slope, curvature, and level).

Our static analysis hints that Bitcoin exhibits a high level of independence to shocks from the yield curve components and from the main fiat currencies. These results imply that Bitcoin might offer hedging property. However, the dynamic analysis shows that Bitcoin is far from being isolated from other currencies or the yield curve components, even more so in stressful times. The results are consistent for both the exchange rates and the volatility of exchange rates. Notably, our time-varying analysis indicates that the connectedness increases due to policy shocks, such as the removal of the cap on the Swiss Franc against the Euro, the political uncertainty created by BREXIT, and the systemic crisis of COVID-19, suggesting that Bitcoin is far from being counted as a safe-haven asset. The results support previous studies that argued the limited risk reduction capability of Bitcoin and its failure to act as a safe-haven asset.

These main findings may have implications for financial market participants and policymakers. Monitoring the connectedness across time may assist them with their hedging and risk management as part of their investment decisions, and improving their portfolio selections and asset allocation. Our findings underscore the importance of accounting for a dynamic methodology to comprehend the connectedness between Bitcoin, the main fiat currencies, and the yield curve components.

The remainder of this paper is structured as follows. Section 2 presents a literature review of relevant papers. Section 3 offers a description of the sample. Section 4 describes the methodology, the data sources, and descriptive analysis. Section 5 discusses the empirical results, and Sect. 6 concludes the paper.

## Literature review

A growing stream of studies deals with the relationship between Bitcoin and conventional assets, which is part of the gradual integration of Bitcoin and cryptocurrencies into international financial markets.^2^ This growth is coupled with an increased interest in Fintech and the increased role of opinion dynamics in finance.^3^ Although several works are focusing on the interplay between Bitcoin and other traditional currencies, they are still rare. Recent developments, such as BREXIT and financial crises, that promote uncertainty or even distrust in the conventional monetary or sovereign systems require more examinations of these cryptocurrencies. For example, Dao et al. (2019) showed that BREXIT is associated with an increase in the correlations among safe-haven currencies (CHF and JPY) and a decrease in the correlation between GBP and EUR, which were directly involved in the BREXIT decision. For the latter two currencies, they also found a significant decrease of 64% in the transmission of volatility between GBP and EUR after the BREXIT decision. Meanwhile, Fatum and Yamamoto (2016) analyzed the behavior of currencies during the 2008 global financial crisis, and they reported that JPY is the safest currency among the major currencies (USD, JPY, CHF, EUR, GBP, SEK, and CAD). More recently, one of the few studies including Bitcoin and conventional currencies established Bitcoin as a hedge against the CHF, CAD, and JPY (Urquhart and Zhang 2019). They also reported that Bitcoin is a safer haven during turmoil than the CHF, CAD, and GBP. Zeng et al. (2020) showed that the connectedness between Bitcoin and conventional assets, such as stocks, oil, and gold, is weak. They also demonstrated an asymmetric pattern in spillovers concerning positive and negative returns in the Bitcoin market. Our paper extends these examinations regarding these major currencies and analyzes their connectedness with the yield curve components and Bitcoin.

The second research stream that our paper extends is whether Bitcoin is integrated with or insulated from conventional assets. As we will discuss, the empirical evidence of Bitcoin being a safe haven is debatable. Therefore, our work adds a different perspective on testing Bitcoin’s risk reduction capabilities.

Baur and Lucey (2020) indicated three roles that an asset can play with respect to risk: a diversifier, a hedge, or a safe haven. A diversifier is an asset that usually has a positive correlation with another asset. In contrast, a hedge is an asset that usually correlates negatively with another asset or is uncorrelated with it. Finally, a safe haven is an uncorrelated or negatively correlated asset with another asset during stressful times. Subsequent studies have attempted to study the role of Bitcoin and other cryptocurrencies based on these categories, but their findings are mixed. Although several works have supported the ability of Bitcoin to serve as a safe haven, others concluded that Bitcoin has a limited ability to improve the risk–return relationship, and some of them even implied that Bitcoin amplifies risk.

Using a multivariate stochastic volatility model and dynamic conditional correlation (DCC) approach, Kliber et al. (2019) demonstrated that Bitcoin is only a weak hedge in all markets investigated (Japan, Venezuela, China, Estonia, and Sweden) when investment in US dollars is considered. The only exception for Bitcoin being a safe haven is in Venezuela when the investment is in bolivar. Mensi et al. (2020) explored the co-movement of Bitcoin with Islamic stock markets and concluded that the benefits of portfolio diversification with the former vary across time and frequencies. More specifically, they reported that Bitcoin’s contribution is smaller over longer periods. Meanwhile, using the cross-quantilogram approach to test the safe-haven properties of Bitcoin, gold, and commodities, Shahzad et al. (2019) concluded that Bitcoin is a weak safe haven for the MSCI World Stock Market Index and the Chinese Index, but this property depends on time. Wang et al. (2019) utilized the DCC approach to explore the dynamics between weighted and equal cryptoindices created from a pool of 973 cryptocurrencies and 30 equity indices. They indicated that cryptocurrencies are a safe haven for several international indices but only in certain periods. Moreover, the safe-haven property is more pronounced in developed markets and when using larger (in terms of market capitalization) and more liquid cryptocurrencies. Moreover, Umar et al. (2021a, b) studied the connectedness between cryptocurrencies and the technology sector. They documented that the cryptocurrency market is less integrated with the technology sector and thus less exposed to systemic shocks.

However, Smales (2019) ruled out the potential of Bitcoin to act as a safe-haven asset. He claimed that this is due to more volatility, less liquidity, and higher transaction costs of Bitcoin. Klein et al. (2018) also questioned whether Bitcoin could be considered the new gold. They indicated that Bitcoin and gold are entirely different. Specifically, correlations of Bitcoin with conventional assets behave differently from gold. Bitcoin is positively correlated with equity indices in market downturns, suggesting that it is not a safe haven. Baur et al. (2018) showed that Bitcoin is essentially a speculative investment and is not an alternative currency or medium of exchange. Dyhrberg (2016a, b) concluded that Bitcoin has several similarities to gold and the dollar, which implies a hedging property. They argued that Bitcoin can serve as a hedge for equities and against the dollar.

Recent studies also exploit the COVID-19 crisis to explore the risk reduction properties of Bitcoin. Many studies, for example, Conlon and McGee (2020), have examined the potential safe-haven feature of Bitcoin during the COVID-19 crisis. However, they doubted the ability of Bitcoin to serve as a safe haven given its decrease in price along with the S&P 500 index. They also showed that having even a small proportion of Bitcoin in one’s portfolio increases its downside risk. Corbet et al. (2020a, b, c) tested the dynamic correlations between Bitcoin and the Chinese financial markets. They concluded that Bitcoin is not a safe haven. In fact, they argued that it is actually an amplifier of contagion. Moreover, Conlon et al. (2020) examined the safe-haven properties of Bitcoin, Ethereum, and Tether against the most impacted international equity indices during the initial stage of COVID-19—the leading indices in the US, the UK, Italy, Spain, and China. They reported that Bitcoin and Ethereum cannot serve as safe havens for most of these equity indices during the COVID-19 turmoil. Tether does demonstrate a safe-haven property, but it is not consistent across time. Furthermore, Umar and Gubareva (2020) analyzed the impact of COVID-19-induced panic on traditional and cryptocurrencies. They also concluded that cross-currency hedging strategies fail during a systemic event such as the COVID-19 pandemic.

Our final contribution is the inclusion of the three components of the yield curve in our analysis. Doing so contributes to the growing field of studies dealing with the relationships among conventional currencies, Bitcoin, macroeconomic information, and monetary systems (e.g., Nguyen et al. 2019; Corbet et al. 2020a, b). For example, in their exploration of the response of the major cryptocurrencies (Bitcoin, Ethereum, Litecoin, and Ripple) to tightening versus easing monetary regimes, Nguyen et al. (2019) indicated that cryptocurrencies respond to Chinese monetary policies, but not to parallel regimes in the US. Bitcoin and cryptocurrencies have been designed to be detached from any conventional monetary systems; thus, we present the first attempt to explore whether movements in different parts of the yield curve (level, slope, and curvature) are connected to the behavior of Bitcoin’s price. Pyo and Lee (2020) explored the impact of the Federal Open Market Committee (FOMC) and macroeconomic announcements on the behavior of Bitcoin prices. They found that although Bitcoin responds to FOMC announcements, it does not react to macroeconomic announcements, such as the employment rate and the Consumer Price Index. Meanwhile, Lyócsa et al. (2020) showed that Bitcoin’s volatility is not affected by monetary policy announcements in the US or by macroeconomic announcements about budget deficits and inflation.

Two interesting mechanisms may underly the interplay between Bitcoin and the yield curve. If Bitcoin is indeed disconnected from the monetary systems, it should also be insulated from fluctuations in the yield curve. However, the growing popularity of Bitcoin that has resulted in its integration by firms and financial markets could result in increased connectivity.

To summarize, our study extends and contributes to the existing literature in several aspects. First, we extend the examinations done so far in the context of major currencies and their connectedness with the components of the yield curve and Bitcoin. Although several recent papers have examined the relationship between Bitcoin and conventional currencies, they are still scarce, and we are motivated to extend the empirical evidence in this respect. In addition, we are not aware of any study that has included the components of the yield curve, major currencies, and Bitcoin in its assessments about their connectedness.

Second, the general debate about Bitcoin’s property as a safe haven is far from being resolved. Although a rapidly growing stream of studies was conducted on this issue, the evidence supporting Bitcoin’s ability to reduce risk is still vague. Therefore, we contribute to the general discussion in the literature regarding Bitcoin’s risk reduction capabilities for the variables examined.

Lastly, our study also contributes to the aforementioned studies dealing with Bitcoin, conventional currencies, macroeconomic information, and monetary systems by tracking the interplay between the movements of the yield curve components and Bitcoin’s performance.

## Data and descriptive analysis

Our sample period spans from May 11, 2010, to November 26, 2020, based on the availability of the matched series data. As the first step, we construct the components of the yield curve. Toward this end, we utilize the zero-coupon sovereign yield for the US with 15 monthly maturities, including 3, 6, 9, 12, 24, 36, 48, 60, 72, 84, 96, 108, 120, 240, and 360 months. The sovereign yield data are retrieved from Bloomberg. We use Diebold and Li’s (2006) methodology to decompose these zero-coupon yields into the term structure components of the yield curve: level, slope, and curvature. The decomposed components of the yield curve are not stationary; therefore, we take the first difference of each series to ensure stationarity and confirm it with augmented Dickey– Fuller (1979) (ADF) tests.^4^ For brevity, we do not report the ADF test results, but they are available upon request.

Next, we obtain the historical exchange rates denominated in US dollars for each fiat currency (CAD, CHF, EURO, JPY, and GBP) and Bitcoin from Bloomberg. The exchange rates are not stationary, and therefore, we compute the first difference of levels for each currency. We confirm stationarity by employing the ADF tests. Lastly, we obtain the historical volatility of each fiat currency and Bitcoin from Bloomberg. Here again, the raw volatility series obtained from Bloomberg is non-stationary. Consequently, we take the first differences to ensure stationarity and confirm it with the ADF tests.

Table 1 presents the descriptive statistics of the exchange rates of Bitcoin, the fiat currencies, and the yield curve components. We note the significantly high average price and standard deviation of Bitcoin compared to the other fiat currencies, which we attribute to the strong demand for Bitcoin in recent years. Figure 4a of Appendix depicts the level and the first difference of the exchange rates and the components of the yield curve. Similarly, Fig. 5 of Appendix presents the level and the first difference of the historical volatility of the exchange rate for Bitcoin, the fiat currencies, and yield curve components.

**Table 1:** Descriptive statistics for the key variables

	Bitcoin	CAD	CHF	Euro	GBP	JPY	Level	Slope	Curvature
Mean	2925.6790	0.8499	1.0528	1.2143	1.4499	0.0099	3.5837	− 2.9394	− 2.5848
Median	564.6300	0.7921	1.0400	1.1787	1.4903	0.0093	3.2972	− 2.9578	− 1.7097
Maximum	18,944.8600	1.0601	1.3872	1.4830	1.7166	0.0132	6.7863	0.2069	1.0043
Minimum	0.2000	0.6859	0.9707	1.0388	1.1485	0.0080	0.9351	− 6.7422	− 8.6862
Std. Dev	4057.3760	0.1090	0.0512	0.1113	0.1504	0.0015	1.2458	1.7604	2.2239
Skewness	1.3181	0.4478	1.4832	0.4169	− 0.0711	0.9944	0.5190	− 0.1926	− 0.4778
Kurtosis	3.7336	1.5228	7.0233	1.8320	1.4597	2.6009	2.9062	2.0358	1.8961
Jarque–Bera	817.77	325.90	2728.71	224.92	261.31	449.35	118.61	117.74	232.82
Probability	0	0	0	0	0	0	0	0	0
Observations	2621	2621	2621	2621	2621	2621	2621	2621	2621

The table reports the descriptive statistics of the exchange rates of five major safe haven currencies (CAD = Canadian Dollar, CHF = Swiss Franc, GBP = Great British Pound, JPY = Japanese Yen) and Bitcoin as well as the three components of the US yield curve (Level, Slope and Curvature)

## Methodology

Our empirical framework is composed of two methodological steps. In the first step, we use the dynamic modification of Diebold and Li (2006) to Nelson and Siegel’s (1987) model by using maximum likelihood and Kalman filter. Then, we estimate the three US yield curve components: level, slope, and curvature. Notably, the use of Nelson–Siegel (1987) approach for estimating the yield curve components conveys several advantages. Among others, it offers parsimonious estimates for the yield curve components, does not impose arbitrage-free restrictions, and fits any type of yield curve. Several studies in the literature (e.g., Diebold et al. 2006; Diebold and Li 2006; Vicente and Tabak 2008; Yu and Salyards 2009) also argued that the Nelson–Siegel model suggests a superb prescience and predictability of the yield curve. Consequently, this approach became a preferable approach among researchers.

Subsequently, we follow the methodology of Diebold and Yilmaz (2009, 2012, 2014) for a dynamic track of the connectedness between the desired system variables. In the following subsections, we supply a concise description of each step.

### Estimating the yield curve components

The groundwork of the Nelson–Siegel model inspired Diebold and Li (2006) to suggest a dynamic estimation for the level, slope, and curvature of the yield curve. They stated that the three coefficients in the Nelson–Siegel curve are latent level, slope, and curvature factors. The following is the state-space representation of Diebold and Li (2006):

1 $${{\varvec{y}}}_{{\varvec{t}}}\left({\varvec{\tau}}\right)={\left(\begin{array}{ccc}1& \left(\frac{1-{e}^{-\lambda {\tau }_{1}}}{\lambda {\tau }_{1}}\right)& \left(\frac{1-{e}^{-\lambda {\tau }_{1}}}{\lambda {\tau }_{1}}-{e}^{-\lambda {\tau }_{1}}\right)\\ 1& \left(\frac{1-{e}^{-\lambda {\tau }_{2}}}{\lambda {\tau }_{2}}\right)& \left(\frac{1-{e}^{-\lambda {\tau }_{2}}}{\lambda {\tau }_{2}}-{e}^{-\lambda {\tau }_{2}}\right)\\ \begin{array}{c}\vdots \\ 1\end{array}& \begin{array}{c}\vdots \\ \left(\frac{1-{e}^{-\lambda {\tau }_{N}}}{\lambda {\tau }_{N}}\right)\end{array}& \begin{array}{c}\vdots \\ \left(\frac{1-{e}^{-\lambda {\tau }_{N}}}{\lambda {\tau }_{N}}-{e}^{-\lambda {\tau }_{N}}\right)\end{array}\end{array}\right)}{x}_{t}+{u}_{t}, {u}_{t}\sim N\left(0,R\right)$$

Moreover, the transition equation is

2 $$\widetilde{{x_{t} }} = ~\Gamma \tilde{x}_{{t - 1~}} + ~\eta _{t} ~\eta _{t} \tilde{N}\left( {0,G} \right)$$

where ${y}_{t}\left(\tau \right)$ denotes an N × 1 dimensional vector for yield rates, ${u}_{t}$ denotes an N × 1 vector of error terms. ${x}_{t}=[{L}_{t}, {S}_{t},{C}_{t}]$ is a 3 × 1 dimensional vector containing the latent factors of the yield curve with ${L}_{t}$ stands for the level factor,${S}_{t}$ denotes the slope factor, and ${C}_{t}$ is the notation for the curvature factor. In the subsequent transition equation, ${\sim }{{x}_{t}} \,=\, {x}_{t}- {\tilde{x }}_{t-1}$ represents the matrix of demeaned time-varying shape parameters, $\Gamma$ denotes the dynamic relationship across shape parameters, ${\eta }_{t}$ denotes the error vector with dimension 3 × 1. ${\eta }_{t}$ and ${u}_{t}$ are assumed to be independent. $G$ is a diagonal matric with dimension N × N. Lastly, $R$ denotes a 3 × 3 dimensional variance–covariance matrix.^5^

### The Diebold and Yilmaz connectedness approach

Estimating and examining the spillover and connectivity dynamics of variables in a certain system (e.g., wavelets, dynamic copulas with regime-switching and global vector autoregression model) may have different types of practices and approaches. We adopt the novel method of Diebold and Yilmaz (2009, 2012, 2014) that is a well-common approach and enhances the comparability of our results.^6^

Diebold and Yilmaz (2009, 2012, 2014) approach is based on the well-known VAR model by Sims (1980), which has been a main tool by researchers and economists. In essence, they suggested an interesting arrangement and use of the forecast error variance decomposition (FEVD) as an interpretation for the connectivity between the variables of a certain system. Given that the FEVD provides information about the degree to which a counterpart variable explains a future variation in a certain variable in the system, they managed to construct a connectedness off-diagonal matrix for the variables of the system. Accordingly, one can observe the proportion of a certain variable’s future values that were originally from other variables in the system and have an assessment as to spillover effects “TO” and “FROM” each variable.

Following Diebold and Yilmaz (2009, 2012, 2014), we start by considering a *k*th order, N variable VAR:

3 $${y}_{t}={\sum }_{k=1}^{K}{\Theta }_{k}{y}_{t-k}+{\varepsilon }_{t}$$

where ${y}_{t}={y}_{1t , }{y}_{2t , }\dots ,{y}_{Nt}$ denotes the vector of endogenous variables that are assumed to be connected, ${\Theta }_{k}, k=1,\dots ,k$ denotes the N × N parameter matrices, and ${\varepsilon }_{t}\sim (0,\Sigma )$ is the vector of i.i.d. error terms.

Equation (3) describes that each variable in the system is explained by its own lagged values and the rest of system variables. Under the assumption of covariance stationarity, the moving average representation of Eq. (1) can be given as ${y}_{t}= {\sum }_{p=0}^{\infty }{A}_{p}{\varepsilon }_{t-p}$, where ${A}_{p}$ denotes the N × N coefficient matrices, such that ${A}_{p}= {\Theta }_{1}{A}_{p-1}+{\Theta }_{2}{A}_{p-2}+\dots +{\Theta }_{p}{A}_{p-l},$ where ${A}_{0}$ is the N × N identity matrix and ${A}_{p}=0$ for all p < 0.

The traditional techniques like the Cholesky factorization for estimating orthogonal innovations are sensitive to variable ordering. Therefore, we follow the solution of Koop et al. (1996) and Pesaran and Shin (1998), who proposed a generalized forecast error variance decomposition (GFEVD) that is invariant to ordering. The *H*-step generalized variance decomposition matrix is given as D^gH^ =  $[{d}_{ij}^{gH}]$, where ${d}_{ij}^{gH}$ is

4 $${d}_{ij}^{gH}=\frac{{\sigma }_{jj}^{-1}{\sum }_{h=0}^{H-1}({e}_{i}^{\mathrm{^{\prime}}}{A}_{h}\sum {e}_{j}{)}^{2}}{{\sum }_{h=0}^{H-1}({e}_{i}^{\mathrm{^{\prime}}}{A}_{h}\sum {A}_{h}^{\mathrm{^{\prime}}}{e}_{j})}$$

where Σ denotes the (estimated) variance matrix of the error vector ε, *e*_*j*_ is a selection vector with *j*th element unity and zeros everywhere, and *σ*_*jj*_ is the *j*th diagonal element of Σ*.* Sums of forecast error variance contributions (i.e., row sums of *D*^*g*^) are not necessarily unified, as shocks are not necessarily orthogonal in the GFEVD environment. Hence, we base our generalized connectedness indexes not on *D*^*g*^, but rather on ${\tilde{D }}^{g}=[{\tilde{d }}_{ij}^{g}]$, where ${\tilde{d }}_{ij}^{g}=\frac{{d}_{ij}^{g}}{{\sum }_{j=1}^{N}{d}_{ij}^{g}}$. By construction, ${\sum }_{j=1}^{N}{\tilde{d }}_{ij}^{g}=1$ and ${\sum }_{i,j=1}^{N}{\tilde{d }}_{ij}^{g}=N$. Using ${\tilde{D }}^{g}$, we can immediately calculate generalized connectedness measures.

To understand the connectedness and relationships between the variables of a certain system, Diebold and Yilmaz (2014) constructed a connectedness matrix, as shown in Table 4. The upper-left N × N block is the so-called “variance decomposition matrix.” It is denoted by ${D}^{H}=\left[{d}_{ij}^{H}\right],$ where ${d}_{ij}^{H}$ represents the *H*-step-ahead variance decomposition component of variable *i* due to shocks in variable *j*. The connectedness table merely supplements ${D}^{H}$ with a rightmost column containing row sums, a bottom row containing column sums, and a bottom-right element containing the grand average, in all cases, for *i ≠ j*. The off-diagonal entries of ${D}^{H}$ are the parts of the N forecast error variance decompositions of relevance from a connectedness perspective; they measure the pairwise directional connectedness. In particular, the gross pairwise directional connectedness from *j* to *i* is given as follows:

5 $${C}_{i\leftarrow j}^{H}={d}_{ij}^{H}$$

Notably, the connectedness to and from may be asymmetric in the sense that ${C}_{i\leftarrow j}^{H}\ne {C}_{j\leftarrow i}^{H}$; hence, ${N}^{2}-\mathrm{N}$ different connectedness measures potentially exist. To obtain the net role of each variable relative to a certain other counterpart variable, that is, whether it functions as a transmitter or a receiver, we compute the net pairwise directional connectedness as follows:

6 $${C}_{ij}^{H}={C}_{j\leftarrow i}^{H}-{C}_{i\leftarrow j}^{H}$$

Similarly, one can be interested in the total connectedness FROM all variables as a whole to variable *i*. In this case, the connectedness measure is computed as follows:

7 $$C_{{i \leftarrow \bullet }}^{H} = \mathop \sum \limits_{{\begin{array}{*{20}c} {j = 1} \\ {j \ne i} \\ \end{array} }}^{N} d_{{ij}}^{H}$$

Alternatively, the total connectedness of a single system variable *j* TO the system can be measured by

8 $$C_{{ \bullet \leftarrow j}}^{H} = \mathop \sum \limits_{{\begin{array}{*{20}c} {i = 1} \\ {j \ne i} \\ \end{array} }}^{N} d_{{ji}}^{H}$$

In this respect, the net total directional connectedness between a single system variable *i* and the system as a whole is defined as

9 $$C_{i}^{H} = C_{{ \bullet \leftarrow i}}^{H} - C_{{i \leftarrow \bullet }}^{H}$$

To have a general assessment of the degree of connectedness in the defined system, we use the summation of the values in the “FROM” column and “TO” row to measure the total connectedness:

10 $${C}^{H}=\frac{1}{N}{\sum }_{\begin{array}{c}i,j=1\\ j\ne i\end{array}}^{N}{d}_{ij}^{H}$$

The total connectedness measure in the system is bounded in the interval of 0 and 1. The lower bound represents a variable system according to which no spillover risk exists, whereas the upper bound of 1 applies to the case of a full connectedness according to which all risks are generated by the system variable interactions.

Following Zeng et al. (2020), Diebold and Yilmaz (2009, 2012, 2014), and others, we set the connectedness horizon, H = 10. Diebold and Yilmaz pointed that a 10-day look-ahead may be in line with the 10-day value at risk (VaR) required under the Basel accord. Naturally, one can use different lengths from 10-day look-ahead, which matches its own risk management preferences. We also use a rolling window of 200 days (approximately nine months) to evaluate the dynamic connectedness measures.^7^

## Empirical results and discussion

### Static full sample connectedness analysis

We start our analysis by discussing the connectedness of the system that contains nine variables of interest ‒ the three components of the US yield curve, Bitcoin, and the five major safe-haven fiat currencies (i.e., Euro, JPY, CAD, CHF, and GBP). We discuss the connectedness of the exchange rates and the yield curve components first, followed by the connectedness of the exchange rate volatility and the yield curve components.

The results in Table 2 show the connectedness among the exchange rates of the safe-haven currencies, Bitcoin, and the yield curve components. The total connectedness index (TCI in the right bottom corner) that expresses the overall degree of connectivity in the system is 39.92%. This result implies that nearly 40% of the variation in the system variables may be explained by their joint dynamics. To delve deeper and see the contribution of each variable to the connectedness of the system, we look at the second last row “TO.” We note that the slope (66.46%) and the level (62.09%) components of the yield curve are the leading contributors of spillover TO the system, followed by the Euro (57.57%). The spillover received by each variable from the system is depicted in the last column “FROM.” Here again, we note that the yield curve’s level and slope are the major recipients of spillover from the system (56.15% and 57.99%, respectively). Among the safe-haven currencies, the Euro receives the most spillovers from the system.

**Table 2:** Static connectedness of the exchange rates and components of the yield curve

Variable	BITCOIN	EURO	GBP	JPY	CHF	CAD	LEVEL	SLOPE	CURVATURE	FROM
BITCOIN	99.16	0.03	0.23	0.01	0.02	0.3	0.13	0.05	0.06	0.84
EURO	0.03	51.54	16.85	4.46	14.98	10.69	0.28	0.26	0.92	48.46
GBP	0.21	19.4	59.64	1.79	6.51	11.37	0.36	0.28	0.45	40.36
JPY	0.03	5.21	1.96	60.38	7.17	0.52	8.09	8.09	8.54	39.62
CHF	0.02	17.41	6.63	7.14	59.95	3.18	0.88	1	3.78	40.05
CAD	0.18	13.58	12.11	0.49	3.5	64.42	2.91	2.59	0.21	35.58
LEVEL	0.03	0.45	0.32	5.74	0.81	1.98	43.85	38.9	7.92	56.15
SLOPE	0.02	0.36	0.22	5.62	0.88	1.69	38.2	42.01	11	57.99
CURVATURE	0.03	1.12	0.45	8.01	3.6	0.54	11.23	15.28	59.73	40.27
TO	0.54	57.57	38.77	33.26	37.46	30.27	62.09	66.46	32.88	TCI
NET	− 0.3	9.11	− 1.59	− 6.35	− 2.59	− 5.31	5.94	8.47	− 7.39	39.92

The table presents the static analysis of the full sample of the exchange rates of Bitcoin and safe haven currencies (EURO, GBP = Great British Pound, JPY = Japanese Yen, CHF = Swiss Franc, CAD = Canadian Dollar) and the components of the US yield curve (Level, Slope and Curvature). ‘From’ (last column) mean spillover of the system of all other variables to the variable. ‘TO’ (second to last row) shows spillover from each variable to the system of all other variables. Net (last row) shows the net directional spillover of each variable. TCI (bold right bottom corner) is the total connectedness index of the system of all variables

Although the “TO” and “FROM” analyses highlight each variable’s contribution to the system, net transmitters and net receivers of spillovers must also be distinguished. This information is presented in the last row of Table 2 (“NET”), showing each variable’s net spillover. A positive value implies that the variable is a net transmitter, whereas a negative value implies that the variable is a net receiver of spillovers. Note that the level and slope of the yield curve along with the Euro are net transmitters of spillovers, whereas all the other variables are net receivers of spillovers.

Interestingly, all three components of the US yield curve or other currencies have negligible influence on Bitcoin. Most variations in the system are generated by the traditional currencies and yield curve components. Moreover, Bitcoin’s independence is evident in terms of Bitcoin contribution TO or FROM the system. Bitcoin contributes only 0.54% of the total variation TO the system and absorbs only 0.84% FROM the system. Consequently, the net spillover of Bitcoin is also negligible at ‒0.3%. Therefore, we might argue that Bitcoin exhibits risk diversification attributes and may hedge against changes in other safe-haven currencies or fluctuations in the yield curve. However, investors should keep in mind that these results also imply that, as a single asset class, Bitcoin is heavily exposed to its idiosyncratic shocks. For the other currencies, relatively larger spillover exists TO and FROM the yield curve components and within the currencies themselves.

**Fig. 1:**
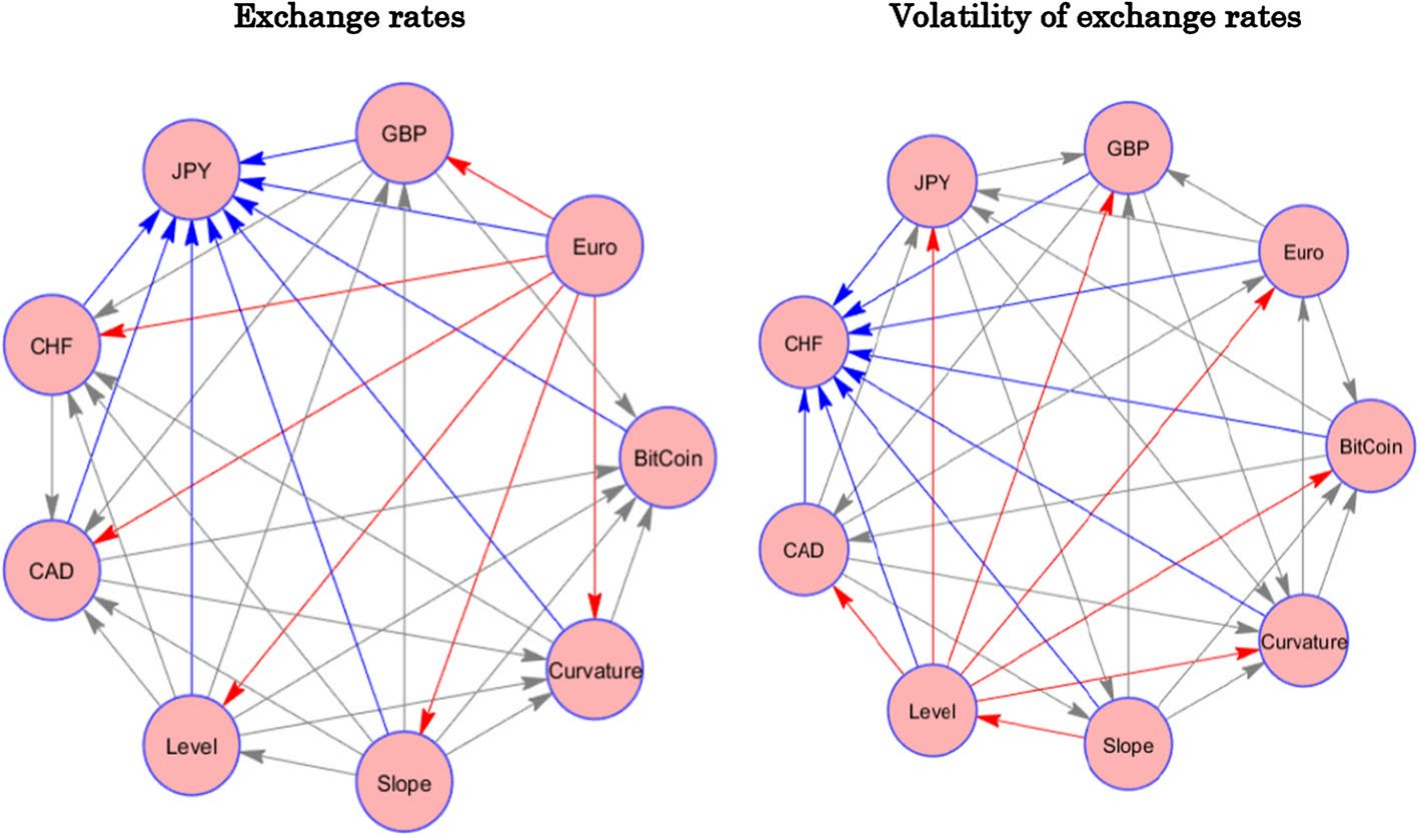
Pairwise Static Net Connectedness of Components of the yield Curve with the Exchange Rates and the Volatility of the Exchange Rates. *Notes*: The figures provide a graphical illustration of the network connectedness of the system consisting of the yield curve components (level, slope, and curvature), Bitcoin and safe haven currencies (EURO, GBP = Great British Pound, JPY = Japanese Yen, CHF = Swiss Franc, CAD = Canadian Dollar). The left-hand (right-hand) graph shows the network connectedness in terms of exchange rates (volatility of exchange rates). Arrows indicate the net directional connectedness between two variables in the system with a one-way direction arrow. The source of the arrow shows the transmitter, and the edge of the arrow shows the receiver of the spillover. More arrows mean a more influential variable in the connectedness. A red font arrow means that the variable has the largest transmitter of pairwise spillovers, while a blue means largest receiver of spillovers

Next, we look at the pairwise spillover. To identify the net transmitters and net receivers of spillovers on a pairwise basis, we resort to network analysis, which enables us to easily analyze the pairwise connections of net transmitters and receivers of spillover. The left-hand panel of Fig. 1 presents the net pairwise connectedness patterns and relationships for the yield curve components, and the exchange rates of Bitcoin and the fiat currencies. The source of the arrow indicates the transmitter of the spillover, whereas the edge of the arrow shows the receiver of the spillover for that particular pair. Looking at the left-hand illustration, we notice that the Euro is the dominant transmitter of shocks to other variables in the system (red lines). Meanwhile, the Japanese Yen is the most prominent recipient of spillover from all other variables (blue lines). Although Bitcoin seems to be also a main recipient of spillovers, as mentioned earlier, the relative magnitude of spillovers for Bitcoin is negligible.

We extend our analysis and discuss the connectedness of the yield curve components and the volatility of the exchange rates of Bitcoin and the fiat currencies. We report these results in Table 3. Overall, the results for volatility connectedness exhibit similar patterns to those for the exchange rate connectedness. The TCI for the yield curve and volatility series is 25.79%, which is lower than the connectedness in terms of the exchange rates but is still sizable. Here again, we note that the slope and level of the yield curve are the most pronounced transmitters, where the curvature is the highest receiver of shocks. In addition, the Euro and the CAD are the net transmitters. All other currencies are net receivers. Regarding the magnitude of the spillovers, Bitcoin and the Swiss Franc exhibit the lowest magnitude of “TO” and “FROM” spillovers for volatility. The lower magnitude of connectedness may be attributed to their potential diversification benefits.

**Table 3:** Static connectedness of the volatility of the exchange rates and components of the yield curve

Variable	BITCOIN	EURO	GBP	JPY	CHF	CAD	LEVEL	SLOPE	CURVATURE	FROM
Bitcoin	98.77	0.42	0.02	0.23	0.01	0.09	0.2	0.2	0.06	1.23
Euro	0.07	72.65	9.22	7.85	1.7	8.21	0.05	0.02	0.23	27.35
GBP	0.02	10.04	76.28	7.03	0.14	6.04	0.18	0.19	0.09	23.72
JPY	0.35	8.81	6.49	79.49	0.64	3.48	0.39	0.11	0.24	20.51
CHF	0.32	2.21	0.18	0.66	95.84	0.15	0.19	0.18	0.27	4.16
CAD	0.16	7.36	6.3	2.95	0.12	82.24	0.43	0.22	0.23	17.76
Level	0.13	0.03	0.11	0.12	0.09	0.34	47.99	42.58	8.62	52.01
Slope	0.11	0.02	0.07	0.19	0.09	0.31	41.57	45.73	11.91	54.27
Curvature	0.02	0.19	0.11	0.26	0.18	0.25	12.71	17.36	68.91	31.09
TO	1.19	29.08	22.49	19.29	2.97	18.87	55.72	60.85	21.64	TCI
NET	− 0.04	1.73	− 1.23	− 1.22	− 1.18	1.11	3.7	6.58	− 9.45	25.79

The table presents the static analysis of the full sample of the volatility of the exchange rates of Bitcoin and safe haven currencies (EURO, GBP = Great British Pound, JPY = Japanese Yen, CHF = Swiss Franc, CAD = Canadian Dollar) and the components of the US yield curve (Level, Slope and Curvature). ‘From’ (last column) mean spillover of the system of all other variables to the variable. ‘TO’ (second to last row) shows spillover from each variable to the system of all other variables. Net (last row) shows the net directional spillover of each variable. TCI (bold right bottom corner) is the total connectedness index of the system of all variables

To analyze the pairwise connectedness of the volatility of the exchange rate series and the yield curve components, we look at the right-hand illustration in Fig. 1. The yield curve level exhibits the largest number of pairwise transmission spillovers to other variables in the system, making it the most influential system variable. Meanwhile, the CHF is the most prominent in terms of the number of pairwise spillovers absorbed, although, as aforementioned, its magnitude is relatively low. The curvature of the yield curve is the next dominant receiver of pairwise spillovers in the system. Although the number of links with other system variables is small, the magnitude is substantially higher than with the CHF.

Our static analysis results accord with those of previous studies documenting the weak connectedness between Bitcoin and traditional assets (e.g., Dyhrberg, 2016a; b; Corbet et al. 2018; Trabelsi 2018; Zeng et al. 2020), which can be translated into hedging benefits. The results are also in line with a recent study by Kurka (2020), who found that unconditionally, both spillovers to and from traditional assets (Euro, JPY, US 2-year T-note, S&P 500 stock index, gold, and crude oil) to Bitcoin are low. However, the author also stated that time-conditional effects that remain hidden in the aggregate present a notable temporary transmission of shocks in the system under study. Therefore, the use of the total period may hide certain patterns due to possible structural breaks or changing trends in their connectivity. Hence, the connectedness portion may be different under a dynamic estimation using a rolling window approach.

### Dynamic analysis

Our second step in the connectedness examination for the variables of interest involves a dynamic approach. Diebold and Yilmaz (2009, 2012, 2014) and many other studies have advocated using a rolling window procedure to address several caveats that might be involved with a static approach. These drawbacks include instability, possible structural breaks, non-stationarity, and the effect of outliers in the variables. A dynamic approach is crucial, particularly when the system includes volatile asset classes, such as fiat currencies and Bitcoin. Therefore, using dynamic analysis not only allows us to comprehend the evolution of the connectedness but also provides an important robustness test and a more informative picture. As explained in the methodology section, we follow Diebold and Yilmaz’s methods and other studies that use 200 days as the rolling window.^8^

**Fig. 2:**
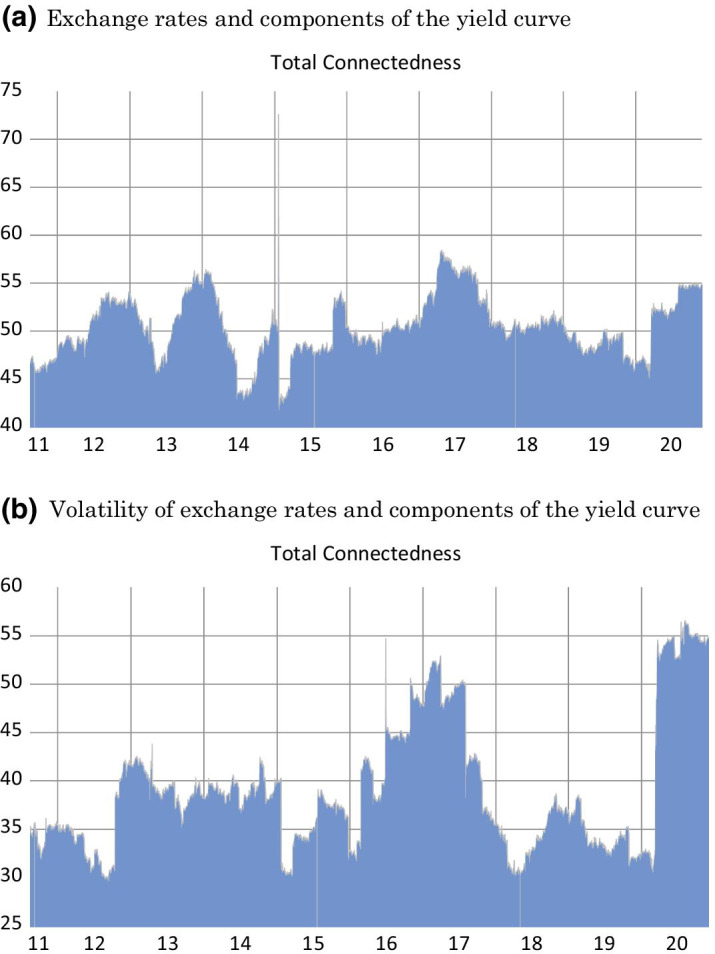
Total Dynamic Connectedness Measures of the System. **a** Exchange rates and components of the yield curve. **b** Volatility of exchange rates and components of the yield curve. *Notes:* The figures present the total directional connectedness of the US yield curve components (level, slope, and curvature), the Bitcoin, and safe haven currencies (Euro, JPY, CAD, CHF, GBP) in a dynamic fashion. The upper figure is illustrates the dynamic connectedness of the yield curve components and the exchange rate of Bitcoin and the safe haven currencies, while the bottom depicts the connectedness of the yield curve components and the volatility of Bitcoin and the safe haven currencies

Figure 2 describes the estimated TCI across time. It contains two graphs depicting the index for the exchange rates (Fig. 2a) and the volatility of the exchange rates (Fig. 2b). We can infer from the graphs that the relationship between the system variables varies across time, justifying the dynamic estimation we conducted. Specifically, one can observe that connectedness is high in some periods. In general, the connectedness index for the exchange rates (Fig. 2a) ranges from 45 to 60%, with an exceptional peak around January 2015. At this point, the Swiss National Bank abandoned the Swiss Franc’s cap on the Euro. We also see an increasing trend in connectedness around the second half of 2016, which might be attributed to the political uncertainty related to BREXIT. The last peak represents the systemic crisis arising from the outbreak of COVID-19. As expected, the time-varying connectedness of the exchange rate volatility and Bitcoin exhibits relatively more variation, with connectedness ranging between 30% and nearly 55% (Fig. 2b). Again, the first main peak is during 2016–2017 due to the political uncertainty in Europe triggered by BREXIT and subsequent elections in the UK and France. The second peak is during the COVID-19 period. These two periods are the only ones in which the connectedness exceeds the value of 50%.

**Fig. 3:**
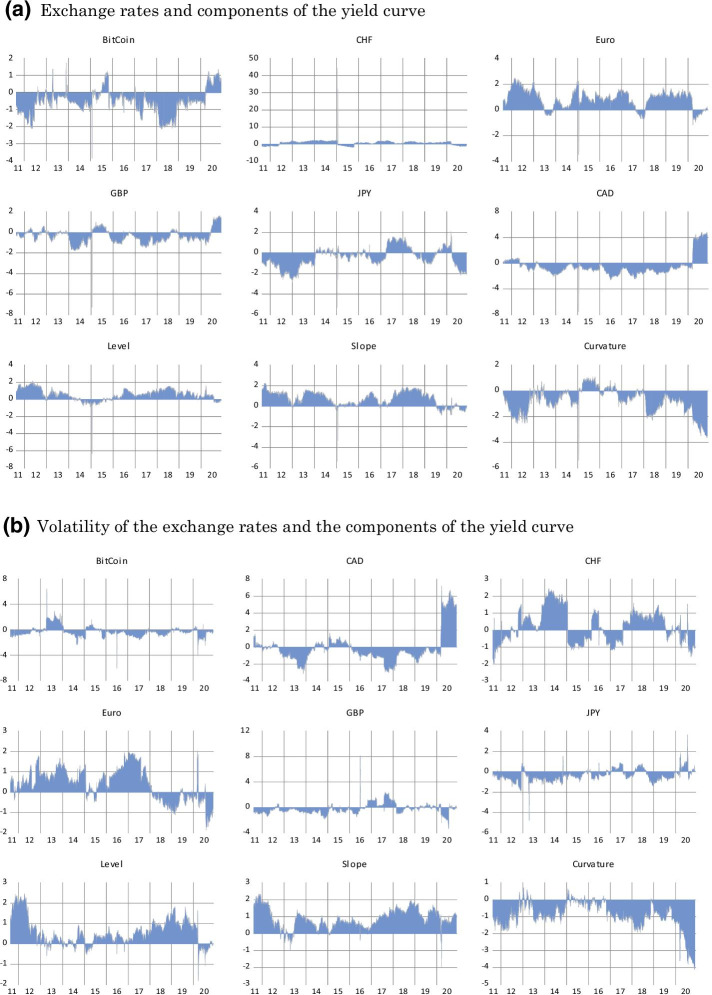
Net Dynamic Connectedness Measure of the System. **a** Exchange rates and components of the yield curve. **b** Volatility of the exchange rates and the components of the yield curve. *Notes*: The figures describe the dynamic net spillover between each variable and the whole system. Positive values indicate a variable X as a net transmitter, while negative values indicate a variable as a net receiver in the system. The top figure represents the spillover of the system of the yield curve components and the exchange rate of Bitcoin and the safe haven currencies. The bottom figure shows the spillover of the yield curve components and the volatility of Bitcoin and the safe haven currencies. The rest of notations as in Fig. 1

**Fig. 4:**
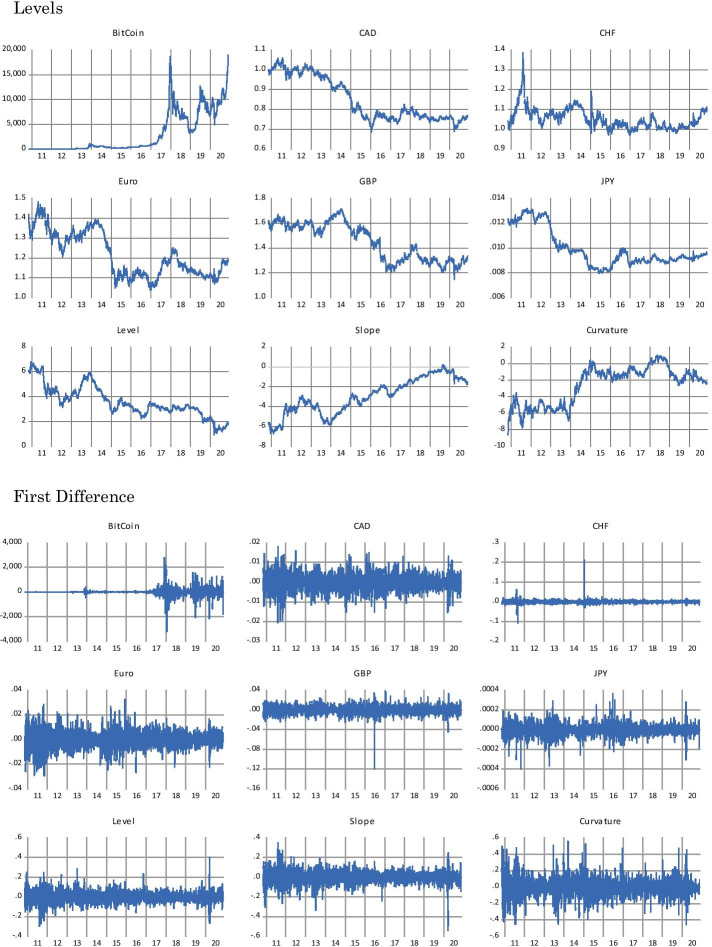
Levels and First Difference of the Exchange Rates and the Components of the Yield Curve. *Notes*: This figure shows the level and first difference of the exchange rate of Bitcoin and the fiat currencies in the top and bottom panels, respectively. The levels and first difference of the components of the yield curve are also reported

**Fig. 5:**
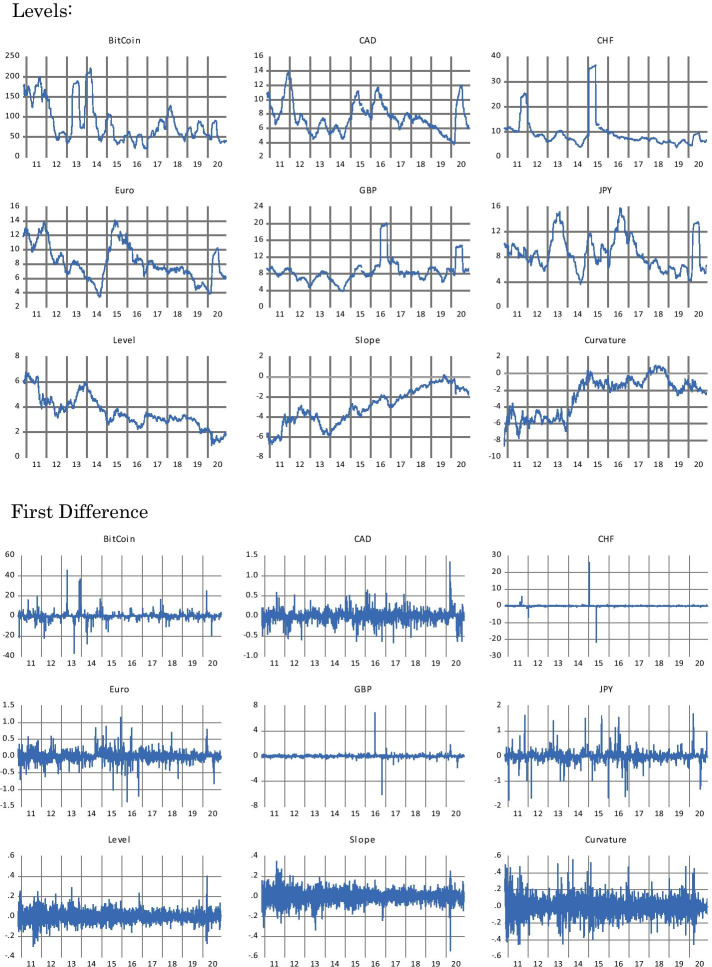
Levels and First Difference of the Volatility of the Exchange Rates and the Components of the Yield Curve. *Notes*: This figure shows the level and first difference of the historical volatility of Bitcoin and the fiat currencies in the top and bottom panels, respectively. The levels and first difference of the components of the yield curve are also reported

Our general dynamic analysis so far shows that the connectedness of the entire system of variables varies over the sample period. However, analyzing the contribution of each variable in the overall connectedness of the system is also equally important. Doing so will help us understand the potential role of Bitcoin in terms of risk reduction. Therefore, we discuss each variable’s dynamic connectedness with the system as a whole, for both the exchange rates (Fig. 3a) and the volatility of the exchange rates (Fig. 3b).

Figures 3a, b provide additional insights into the interactions of each variable with the system.

Figure 3a depicts the net contribution of each variable to all other variables in the system of exchange rates. Positive values indicate that the variable is a net transmitter, whereas negative values imply a net receiver in the system. The results correspond to the general results we observed in the static analysis. The Euro, the level, and the slope of the yield curve are net transmitters. Meanwhile, JPY, GBP, CAD, and Bitcoin are mainly net receivers of spillovers. However, the CAD and Bitcoin become net transmitters in the wake of the COVID-19 period, and the CHF switches roles several times during the sampled years. Bitcoin is a net receiver of spillovers during most of the sample period, with the notable exception of during the COVID-19 pandemic. Furthermore, similar to other currencies, Bitcoin exhibits periods of relatively strong and weak connectedness. Based on these findings, we conclude that its role in terms of risk reduction is also dynamic, varying between a hedge and a diversifier. Given the observed increase in Bitcoin’s connectedness during stressful times, our results also rule out its potential as a safe-haven asset.

Figure 3b. repeats the analysis concerning exchange rate volatility. Similar to the previous results, the Euro is the dominant transmitter of spillovers during most of the sampled period, whereas the CHF exhibits alternating patterns. Most other currencies are net receivers. Here again, we note that the CAD is a major transmitter of spillovers during the COVID-19 period, whereas all other currencies are predominantly net receivers. For the yield curve components, the level and slope are the main transmitters, whereas the curvature is the receiver of spillovers. Lastly, Bitcoin is primarily a receiver of spillovers from the system of the exchange rate volatility and yield curve components. It also still exhibits dynamic connectedness, which strengthens particularly during the COVID-19 period. These results underscore the importance of observing the connectedness level through the lens of a dynamic approach. Doing so allows us to conclude that Bitcoin fails to be a safe-haven asset, and its ability to reduce risk varies between hedging and diversifier roles over time.

**Fig. 6:**
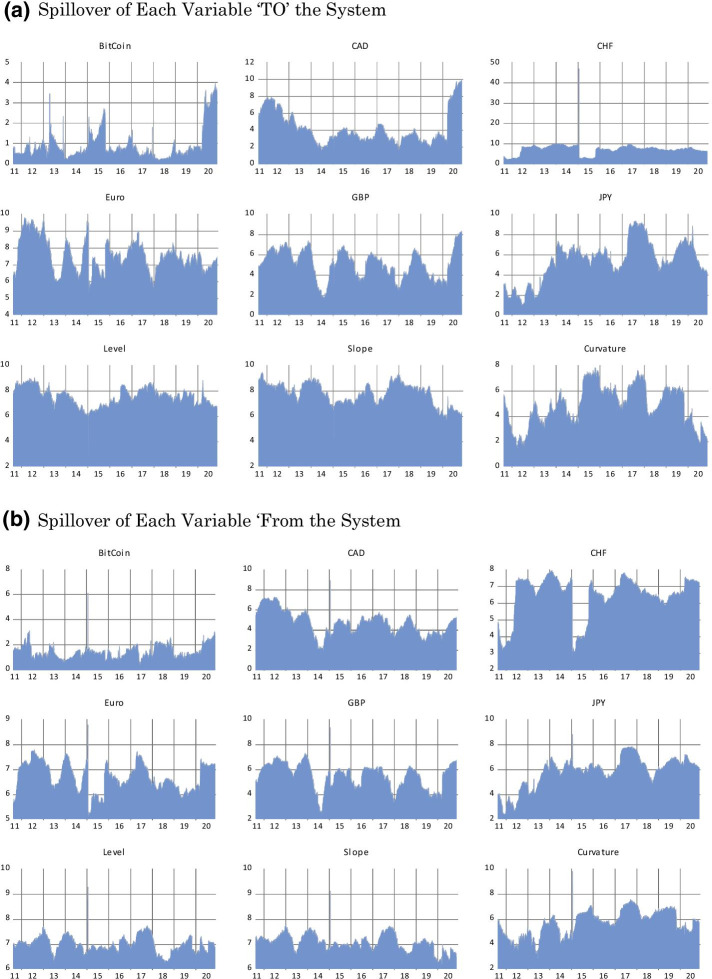
Spillover ‘To’ and ‘From’ the System of the Components of the Yield Curve and the Exchange Rates of the Safe Haven Currencies and Bitcoin. **a** Spillover of Each Variable ‘TO’ the System. **b** Spillover of Each Variable ‘From the System. *Notes*: The figures describe the dynamic ‘TO’ and ‘FROM” spillovers between each variable and the whole system comprising the yield curve components and the exchange rate of Bitcoin and the safe haven currencies. The rest of the notations are as in Fig. 1

**Fig. 7:**
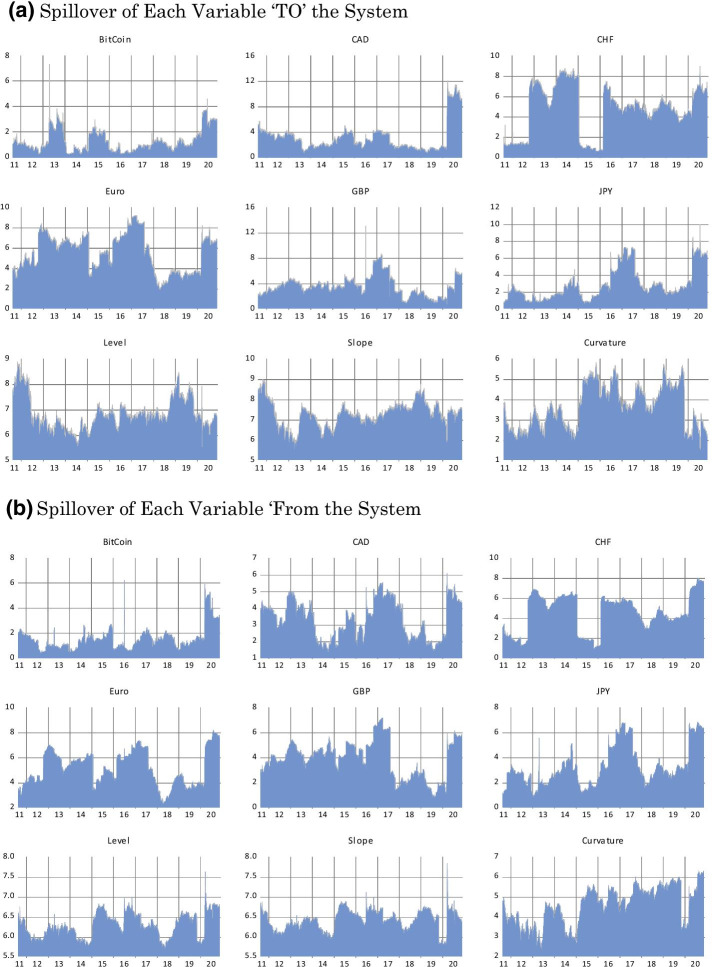
Spillover ‘To’ and ‘From’ the System of the Components of the Yield Curve and the Volatility of the Exchange Rates of the Safe Haven Currencies and Bitcoin. **a** Spillover of Each Variable ‘TO’ the System. **b** Spillover of Each Variable ‘From the System. *Notes*: The figures describe the dynamic ‘TO’ and ‘FROM” spillovers between each variable and the whole system comprising the yield curve components and the volatility of the exchange rate of Bitcoin and the safe haven currencies. The rest of the notations are as in Fig. 1

Further support for the argument of Bitcoin’s failure to act as a safe-haven asset comes from analyzing the dynamic “TO” and “FROM” system spillovers of the exchange rates and the volatility of the exchange rates in Figs. 6 and 7, respectively. Figure 6a depicts the spillover from each listed variable to all other variables in the system, whereas Fig. 6b illustrates the spillover received by each listed variable from all other variables in the system. Bitcoin demonstrates dynamic “TO” and “FROM” spillovers over the sampled period in both the transmission (TO) and absorption (FROM) of shocks. At some points, the spillovers and connectedness are relatively low, but at others, they are considerably higher. For example, Bitcoin peaked during the COVID-19 crisis, resulting in a spike in spillovers for all the currencies. An additional spike coincides with the removal of the cap on the Swiss Franc relative to the Euro in 2015. The Euro and GBP exhibit the greatest variation in both the “TO” and “FROM” spillovers, primarily attributable to the European sovereign debt crisis and the BREXIT crisis.

Lastly, we discuss the “TO” and “FROM” and “Net” spillovers of the volatility of the exchange rates and the yield curve components. Figures 7a,b depict the “TO” and “FROM” spillovers between each variable and the rest of the system variables. Here again, we note the dominance of the Euro in both the “TO” and “FROM” spillovers. Interestingly, all three yield curve components exhibit sizable “TO” and “FROM” spillovers during the sampled period. Although all exchange rates exhibit an increase in the “TO” and “FROM” spillovers during COVID-19, the yield curve components have a slight decrease in the spillovers “TO” the system during this period. Similar to our previous results, although Bitcoin has little connection with the rest of the currencies and the yield curve components in some periods, it seems increasingly connected to the system in stressful times.

An important observation from our dynamic analysis reveals that the hedging capabilities of Bitcoin with other currencies and the yield curve components decrease when we shift from a static to a dynamic analysis. As discussed earlier, the connectedness actually strengthens during turbulent times. Therefore, we can conclude that Bitcoin’s hedging capabilities decline, and the potential of Bitcoin to serve as a shelter or a safe haven is ruled out. These results accord with previous studies arguing against considering Bitcoin a safe-haven asset. For example, Smales (2019) ruled out Bitcoin as a safe-haven asset based on its high volatility, low liquidity and high transaction costs. Similarly, Conlon, Corbet and McGee (2020) conclude that Bitcoin fails to act as a safe-haven asset against major international equity indices. Our findings also support Urquhart and Zhang’s (2019) findings that Bitcoin is a hedge against CHF, CAD, and JPY, but contradict their findings of Bitcoin as a safe haven during turmoil versus the CHF, CAD, and GBP.

## Summary and conclusions

This paper explores the connectedness between the major forex currencies (the CAD, CHF, EURO, JPY, and GBP), Bitcoin, and the components of the US yield curve (level, slope, and curvature). We use Diebold and Li’s (2006) modification of the classic model of Nelson and Siegel (1987) to estimate the yield curve’s level, slope, and curvature. We then employ Diebold and Yilmaz’s (2009, 2012, 2014) connectedness framework to measure the static and dynamic connectedness between the three latent factors of the yield curve, Bitcoin, and the major fiat currencies. The paper estimates the inter-connectedness of the variables in both the exchange rates and the volatility of the exchange rates.

We contribute to the literature dealing with major currency interaction with both the yield curve and the dominant cryptocurrency, the Bitcoin. Given the growing popularity of Bitcoin among individuals, investors, and companies, we present an important attempt to track its potential bidirectional interplay with major currencies. In addition, we extend the examinations to include Bitcoin’s relationship with the yield curve components and reveal whether Bitcoin is connected or isolated from major currencies and conventional monetary assets.

To determine whether Bitcoin has the property of a safe-haven asset, we took a step forward and employed a dynamic connectedness approach, which can distinguish its connectedness during various phases of the economic cycle covered in our sample period. Our dynamic analysis shows that its connectedness actually strengthens during crises and due to policy shocks. Moreover, we document an increase in connectedness due to the removal of the cap on the Swiss Franc against the Euro, the post-BREXIT political uncertainty, and the COVID-19 induced crisis. These findings from the dynamic analysis support previous studies and confirm that Bitcoin is far from being a safe-haven asset (e.g., Baur et al. 2018; Smales 2019). In volatile times, Bitcoin’s connectedness strengths make it incapable of being a shelter from market turmoil against major currencies nor any component of the yield curve.

Policymakers, financial market participants, and practitioners (e.g., debt and forex investors, economists, and multinational firms dealing in different currencies) can benefit from the results in this study to better understand the dynamics between the yield curve movements, safe-haven fiat currencies, and the Bitcoin. Consequently, it may help them improve their allocation and risk management decisions.

Future works on the topic debated in this paper could be further extended in at least two interesting avenues. First, it would be valuable to explore the system connectedness with other leading cryptocurrencies apart from Bitcoin or extend the system explored to include precious metals from the commodity market. Second, it would be interesting to consider the varying effects of good and bad news on the connectedness of the system.

## Appendix

See Table 4; Figs. 4, 5, 6 and 7.

**Table 4:** Diebold and Yilmaz connectedness matrix

	${\rm {X}}_{1}$	${\rm {X}}_{2}$	$\cdots$	${\rm {X}}_{\rm N}$	${\rm From\,others}$
${X}_{1}$	${d}_{11}^{H}$	${d}_{12}^{H}$	$\cdots$	${d}_{1N}^{H}$	${\sum }_{{\varvec{j}}=1}^{{\varvec{N}}}{d}_{1j}^{H}, j\ne 1$
${X}_{2}$	${d}_{21}^{H}$	${d}_{22}^{H}$	$\cdots$	${d}_{2N}^{H}$	${\sum }_{{\varvec{j}}=1}^{{\varvec{N}}}{d}_{2j}^{H}, j\ne 2$
$\vdots$	$\vdots$	$\vdots$			
${X}_{N}$	${d}_{N1}^{H}$	${d}_{N2}^{H}$	$\cdots$	${d}_{NN}^{H}$	${\sum }_{{\varvec{j}}=1}^{{\varvec{N}}}{d}_{Nj}^{H}, j\ne N$
${\rm TO\,others}$	${\sum }_{{\varvec{i}}=1}^{{\varvec{N}}}{d}_{i1}^{H}$	${\sum }_{{\varvec{i}}=1}^{{\varvec{N}}}{d}_{i2}^{H}$	$\cdots$	${\sum }_{{\varvec{i}}=1}^{{\varvec{N}}}{d}_{iN}^{H}$	$\frac{1}{N}{\sum }_{{\varvec{i}},{\varvec{j}}=1}^{{\varvec{N}}}{d}_{ij}^{H}$
	$i\ne 1$	$i\ne 2$	$\cdots$	$i\ne N$	$i\ne j$

## Footnotes

1 Bitcoin price hits an all-time high of more than $28,000 as of the end of December 2020.

2 For a detailed mapping of the cryptocurrency literature, see Corbet et al. ( 2019).

3 Please see Zha et al. (2021) for a detailed review of opinion dynamics in finance, and Kou et al. (2021) for Fintech investments.

4 Importantly, we also use the KPSS test in addition to the augmented Dickey–Fuller test for verifying the stationarity of the variables of interest. The results of KPSS also point on stationary. The results are available upon request.

5 For a detailed discussion and development of the model, refer to Diebold and Li (2006).

6 See, for example, the recent works of Clements and Liao (2020), Fang et al. (2020), Cipollini and Mikaliunaite (2020), Balcilar et al. (2020), Hsu et al. (2020), Zhang and Wang (2020), Umar et al. (2020).

7 Additional robustness test results are available upon request. We also tested different H horizon lengths and longer versus shorter rolling time windows. The results remained qualitatively similar.

8 Notably, the results are qualitatively similar when we split the sample into three and four parts. For brevity, we do not report them here.
